# IN-HOSPITAL NURSE CARE CONTINUITY: DOES IT MATTER?

**DOI:** 10.1093/geroni/igz038.2711

**Published:** 2019-11-08

**Authors:** Orly Tonkikh, Anna Zisberg, Efrat Shadmi

**Affiliations:** 1 University of Haifa, Haifa, Israel; 2 The Cheryl Spencer Department of Nursing Faculty of Social Welfare and Health Science, University of Haifa, Israel, Haifa, Isreal, Israel

## Abstract

In-hospital cognitive decline affects up to 40% of hospitalized older adults and is associated with post-hospitalization worsening of medical and functional status. Studies pointed to the substantial role of the interpersonal relationship between older adults with cognitive impairment and the nurses who care for them. We investigated the association between nursing interpersonal continuity and cognitive outcomes in a cohort of 646 older adults aged 70 or older admitted to internal units for non-disabling conditions. Cognitive decline was defined as at least one point decline in the Short Portable Mental Status Questionnaire from at admission to discharge assessments. Nursing interpersonal continuity was measured using continuity of care index (CoC). CoC assesses the extent of different nurses assigned to take care of each patient during the hospital stay (2 shifts per day) and ranges from 0 (none of the nurses is the same) to 0.4 (highest feasible score according to full time standard shift plan and length of stay (LOS)). Multivariate logistic regression showed that achieving 25% of the highest feasible in-hospital nursing CoC was associated with lower odds of cognitive decline (OR=0.67, 95% CI=0.47-0.97), controlling for age, sex, premorbid activities of daily living status, at admission cognitive status, comorbidities, severity of illness and LOS. This study shows that in-hospital nursing continuity is negatively associated with older adults’ cognitive decline, even in low-continuity levels. Future studies should investigate in-hospital continuity patterns and interventions maintaining continuity in larger and more heterogenic samples.

